# Evaluation of SARS-CoV-2 antigen-based rapid diagnostic kits in Pakistan: formulation of COVID-19 national testing strategy

**DOI:** 10.1186/s12985-021-01505-3

**Published:** 2021-02-13

**Authors:** Umar Saeed, Sara Rizwan Uppal, Zahra Zahid Piracha, Azhar Rasheed, Zubair Aftab, Hafsah Zaheer, Rizwan Uppal

**Affiliations:** 1 Department of Research and Development, Islamabad Diagnostic Center (IDC), F8 Markaz, Islamabad, 44000 Pakistan; 2 Islamabad Diagnostic Center (IDC), G8 Markaz, Islamabad, 44000 Pakistan

**Keywords:** SARS-CoV-2, COVID-19, Rapid diagnostic testing, Nasopharyngeal swab, Saliva, RT-PCR

## Abstract

Rapid diagnosis of SARS-CoV-2 during pandemic enables timely treatment and prevention of COVID-19. Evaluating the accuracy and reliability of rapid diagnostic testing kits is crucial for surveillance and diagnosis of SARS-CoV-2 infections in general population, injection drug users, multi-transfused populations, healthcare workers, prisoners, barbers and other high risk populations. The aim of this study was to evaluate performance and effectiveness of nasopharyngeal swab (NSP) and saliva based rapid antigen detection testing kits in comparison with USFDA approved triple target gold standard real-time polymerase chain reaction. A cross-sectional study was conducted on 33,000 COVID-19 suspected patients. From RT-PCR positive patients, nasopharyngeal swab (NSP) and saliva samples were obtained for evaluation of rapid COVID-19 testing kits (RDT). 100/33,000 (0.3%) of specimens were RT-PCR positive for SARS-CoV-2. Among RT-PCR positive, 62% were males, 34% were females, and 4% were children. The NSP-RDT (Lepu Medical China) analysis revealed 53% reactivity among males, 58% reactivity among females, and 25% reactivity among children. However saliva based RDT (Lepu Medical China) analysis showed 21% reactivity among males and 23% among females, and no reactivity in children. False negative results were significantly more pronounced in saliva based RDT as compared to NSP-RDT. The sensitivity of these NSP-RDT and saliva based RDT were 52% and 21% respectively. The RDTs evaluated in this study showed limited sensitivities in comparison to gold standard RT-PCR, indicating that there is a dire need in Pakistan for development of suitable testing to improve accurate COVID-19 diagnosis in line with national demands.

## Introduction

Severe acute respiratory syndrome (SARS) coronavirus 2 (SARS-CoV-2), belonged to *Betacoronavirus* genera and preferentially infects cells in respiratory tract [1, 2], but its organotropism including brain, conjunctiva, pharynx, lungs, heart, liver and kidneys remains poorly understood [3, 4]. In late 2002, novel coronavirus (SARS-CoV) emerged as an epidemic in Asia, spread worldwide and became serious public health concern internationally [5, 6]. According to World Health Organization (WHO) Emergencies Preparedness Response Report published in 2004, 8096 people around the world were diagnosed with 774 deaths [7]. In December 2019, SARS-CoV-2, a novel coronavirus from same family of SARS-CoV and Middle East Respiratory Syndrome (MERS) coronavirus emerged causing coronavirus disease 2019 (COVID-19) pandemic [8, 9]. The global number of SARS-CoV-2 positive cases were 43,820,929 and have been climbing vigorously. Untill the same period, number of deaths due to COVID-19 were 1,165,189, the worst affected being the United States of America and India [10].

The virus contains four major structural proteins including; matrix core protein (M), nucleocapsid (N), envelop (E), and glycoprotein spike surface (S). It employs spike surface glycoproteins to interact with receptor binding domain, angiotensin‐converting enzyme 2 (ACE2), that is expressed on the epithelial Alveolar type 2 progenitor (AT2) cells of alveoli of lungs, in salivary glands, surface of artery, veins, heart and kidney tissues [11–13]. SARS-CoV enters to host cell via clathrin- and caveolae- independent endocytic pathway [14]. The SARS-CoV-2 generates proteolytically active fragments of RAS (an abbreviation of Rat Sarcoma) protein including Angiotensin II (Ang II) and Angiotensin 1–7 (Ang 1–7) that may activate Angiotensin II receptor type 2 (AT2) receptors to bind with ACE2 and via host cell directed network of G-protein-coupled receptors (GPCRs) ultimately activate c-Jun N-terminal Kinase (JNK) and Janus Tyrosine Kinase (JAK)-Signal Transducer and Activator of Transcription (STAT) biochemical mechanism in host cells and for viral transmission [15].

Recently, a few COVID-19 vaccines were commercially available in developed countries, but are not readily available in several countries across the world possibly due limited production capabilities and funding. Currently, there are more than 50 vaccine candidates of SARS-CoV-2 in trials [16, 17]. Early detection and isolation of infected cases is crucial factor to prevent viral pathogenesis [16]. The nasopharyngeal swab (NPS) followed by real-time reverse-transcription polymerase chain reaction (RT-PCR) of extracted RNA, is recommended gold standard for diagnosis of SARS-CoV-2 and is applied commercially in accordance with WHO protocols [2, 18, 19]. However, during the pandemic, detecting large number of patients via RT-PCR in limited time frame is challenging task and may also involve technical or financial burdens. Rapid, accurate and cost effective diagnosis of SARS-CoV-2 to meet national or international demands for resource limited countries, like Pakistan requires alternative public health containment strategies [19].

Rapid diagnostic tests (RDTs) are user-friendly, cost-effective and safe point-of-care testing; however there is potential concern regarding real-world performance and validation of these assays [20]. The NPS procedure is invasive and may cause bleeding; there are increased chances of SARS-CoV-2 transmission to the healthcare workers [12]. While, saliva specimen collection is non-invasive and can be safely handled outside hospitals [21]. Also, self-collection of saliva samples can reduces risk of SARS-CoV-2 transmission to healthcare workers than NPS [22]. Of note, there was no significant difference in SARS-CoV-2 viral load in NPS or saliva specimens [21].

In saliva or NPS based SARS-CoV-2 RDTs, the challenge is to determine most accurate diagnostic assay without compromising reliability of test results. Analyzing the sensitivity and specificity of most appropriate diagnostic assay or combination of diagnostic assays in comparison to gold standard RT-PCR based test might be helpful in formulating new testing strategy to curtail unprecedented COVID-19 pandemic. There is a dire need in Pakistan to timely develop suitable algorithm that accurately meets national demand for expanded SARS-CoV-2 screening, diagnosis, and treatment. We aimed to evaluate saliva or NPS RDTs based SARS-CoV-2 diagnostic kits with RT-PCR based test to formulate effective testing strategy for diagnosis of SARS-CoV-2 in Pakistani population.

## Materials and methods

To investigate diagnostic accuracy of SARS-CoV-2 antigen in clinical samples (NPS (#20CG2701X, Lepu Medical) or Saliva (#901101, Lepu Medical)) the colloidal gold labeled SARS-CoV-2N protein monoclonal antibody based immunochromatographic rapid test kits were evaluated in comparison with RT-PCR (Bio-rad, CFX96, USA). A cross-sectional study was conducted among 33,000 suspected COVID-19 subjects during the period of 3-10^th^ October 2020 from Islamabad and Rawalpindi cities of Pakistan. After pre-test counseling by trained counselor, the specimens were obtained from SARS-CoV-2 suspected patients with respiratory symptoms and/or fever and international travel history or close contact with SARS-CoV-2 confirmed patients, by trained personnel at Islamabad Diagnostic Center (IDC G8 branch specialized center for COVID-19), Islamabad, Pakistan. Ethical approval was obtained from all 33,000 participants included in this study.

RT-PCR positive cases were pre-selected for evaluation study. All subjects were informed prior to examination and patient consent was obtained. The samples were examined for SARS-CoV-2 by Real-Time PCR after RNA extraction (Auto pure 32 Zybio China). The assay included positive control template and RNA internal extraction control. The triple target genes (design recommended by WHO) were Sarbecovirus E gene, SARS-CoV-2N gene, SARS-CoV-2 RNA-dependent RNA polymerase gene, and detection limit reported by manufacturer was (100) copies/ml. Samples having exponential growth curve and cycle threshhold (Ct) value ≤ 40 were considered as confirmed positive patients. The diagnostic accuracy of NSP and saliva based RDTs testing kits were compared with triple target USFDA approved Seegene kit (#RP10244Y Allplex™ 2019-nCoV Assay, Seegene South Korea) based RT-PCR. The samples with discordant results were repeated. All test procedures were applied according to standard manufacturer protocols. 5 µl template RNA from each sample’s nucleic acid was used with 15 µl of the One-step RT-PCR Master-mix, using following thermocycling conditions consisted of 30 min at 48 °C for reverse transcription, 10 min at 95 °C and 45 cycles of 15 s at 95 °C and 1 min at 60 °C. Seegene kit was stored at -20 freezer, while extracted RNA was stored at -70 deep freezer. These RDTs kits had no cross reactivity with human Coronavirus OC43, Influenza A virus, Influenza B virus, Respiratory Syncytial virus, Adenovirus, Epstein-Barr virus, Measels virus, Cytomegalovirus, Rotavirus, Norovirus, Mumps virus, Varicella Zosyer virus, Human metapneumovirus and Mycoplasma pneumonia. Test kits were stored according to manufacturer’s instructions. The sensitivities were calculated using gold standard RT-PCR results. The study was approved by institutional review board of IDC Pakistan.

## Results

**Fig. 1:**
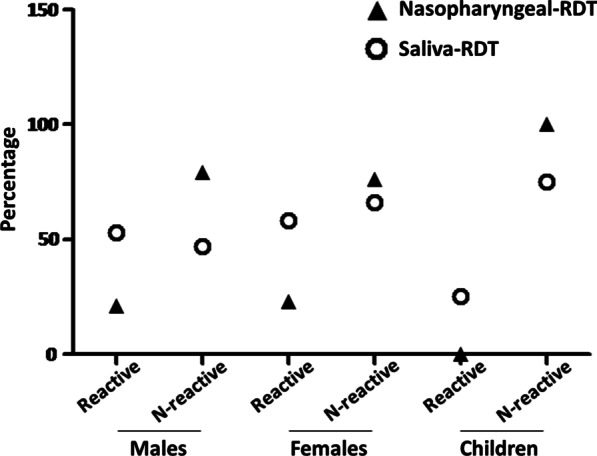
Reactivity percentage of nasopharyngeal and saliva based rapid diagnostic testing kits among several group populations

A total of 33,000 suspected COVID-19 patients were enrolled in this study. Of these, 100 RT-PCR positive (for SARS-CoV-2 RNA) cases were pre-selected for evaluation of COVID-19 antigen kits. The prevalence of SARS-CoV-2 in Islamabad and Rawalpindi regions of Pakistan during period of 3-10^th^ October 2020 was 0.3% (Data analyzed from current study). Among selected subjects, 62% were males, 34% females and 4% were children. The mean age was 47 years (range 6–91). The NSP-RDT analysis revealed a reactive result for SARS-CoV-2 antigen in 52% patients, while remaining 48% were declared non-reactive. The same patient saliva samples (RT-PCR tested positive) were used for saliva based RDT analysis. The data showed 21% reactivity, while remaining 79% showed false-negative results. The NSP-RDT analysis showed 53% reactivity among males, 58% reactivity among females, and 25% reactivity among children. While saliva based RDT revealed 21% reactivity among males, 23% among females, and 0% reactivity in children. False negative results were highly pronounced in saliva based RDT, compared to NSP-RDT. The sensitivity of these NSP-RDT and saliva based RDT were 52% and 21% respectively, as shown in Table 1. The diagnostic accuracy of two RDTs testing kits were compared with triple target USFDA approved Seegene kit based RT-PCR. Among males the reactivity of NSP-RDTs was significantly higher 2.5 fold than Saliva based RDTs (Fig. 1). Same tendency was observed among females (Fig. 1). However among children, only NSP-RDTs showed reactivity (Fig. 1). The sensitivity values of NSP and saliva based assays were 52% and 21% respectively. Average cycle threshold (Ct) values of 100 RT-PCR positive samples taken are shown in Table 1.

**Table 1:** Characteristics of COVID-19 positive patients and assessment of diagnostic accuracy of SARS-CoV-2 rapid diagnostic kits

Samples	Age (Av)	RT-PCR	Ct (Av)	NSP-RDT	Saliva-RDT	Fever	Cough
Male (*n* = 62)	47.48 (19–91)	Positive	28.9	Reactive (*n* = 33)	Reactive (*n* = 13)	Positive	Positive
				N-reactive (*n* = 29)	N-reactive (*n* = 49)		
Female (*n* = 34)	46.47 (20–78)	Positive	28.2	Reactive (*n* = 20)	Reactive (*n* = 8)	Positive	Positive
				N-reactive (*n* = 14)	N-reactive (*n* = 26)		
Children (*n* = 4)	12.25 (6–17)	Positive	32.2	Reactive (*n* = 1)	Reactive (*n* = 0)	Positive	Positive
				N-reactive (*n* = 3)	N-reactive (*n* = 4)		
Total (*n* = 100)	47	Positive	28.5	Sensitivity (52%) Specificity (100%)	Sensitivity (21%) Specificity (100%)	Positive	Positive

## Discussion

In third world countries with acute socio-economic disparities and weaker health system the COVID-19 surveillance and diagnosis of SARS-CoV-2 are serious public health challenges. Italy, followed by United States and South Korea had highest COVID-19 testing per capita compared to rest of the world [23]. According to the WHO recommendations, the SARS-CoV-2 RDTs testing should have minimum sensitivity of ≥ 80% and specificity of ≥ 97% [24]. According to Lepu Medical, among Chinese population, the nasal swab and saliva based RDTs showed 90% sensitivity. The sensitivities in present study (52% for NSP-RDT and 21% for Saliva-RDT) were lower than manufacturers (90%). Similar study from China, reported 68% sensitivity in 208 RT-PCR confirmed nasopharyngeal swab samples [25]. Porte et al., 2020 evaluated fluorescence immunochromatographic SARS-CoV-2 antigen test with nasopharyngeal and oropharyngeal swabs from 127 samples including 82 RT-PCR confirmed patients, with overall sensitivity of 93.9% [26]. Among COVID-19 suspected patients from Kuwait, 83.43% were positive from saliva samples [27]. In present study RT-PCR analysis revealed that the prevalence rate of COVID-19 during 3–10th October in Islamabad/Rawalpindi region of Pakistan was 0.3%, indicating significantly decline pattern compared to near past. Of note, this evaluation was performed during low prevalence of SARS-CoV-2 therefore the performance of antigen-based RDT might vary in different epidemiological conditions.

One of the advantages of this study is the reliability, since same sample materials were used for comparison of RT-PCR *vs* RDTs without possible distribution error from using different specimen. Since SAR-CoV-2 replication is higher in pharynx during initial days after infection and declines later [2, 28], it might be possible that nasopharyngeal swab based antigen test sensitivity is high during initial phase of infection. It is interesting to note that this study was performed at different epidemiological conditions compared to China, and needs further investigation on interracial factors between Pakistani and Chinese populations. Since both rapid tests results were not satisfactory, therefore it is further suggested that combination-test algorithm can be employed for accurate diagnosis of COVID-19.

Viruses are increasing day by day and advanced molecular approaches should be explored to contemplate several signaling pathways and possible host proteins that modulate viral replication [29–33]. The data presented in current study is not only critical for policy making strategic organizations at national level but also answers worldwide call for accurate COVID-19 diagnostic testing. On the basis of this study, formulating advanced testing strategies might reduce technical and financial issues. There are several challenges associated with executing COVID-19 RDT testing strategy including law or policy making, well trained personals, development of quality assurance protocols and resolving technical issues. Therefore, it is important to continuously evaluate RDT based COVID-19 kits in Pakistani populations. Also, correct usage and quality of such COVID-19 RDT must be ensured prior to marketing. Furthermore, it is highly recommended that, on regular basis over time, the government must assure implementation of standard operating procedures for validation of national testing strategies.

## Conclusion

Depending upon the global need for rapid diagnosis of SARS-CoV-2 in limited frame of time, it is necessary to accurately diagnose COVID-19. In resources limited countries like Pakistan, the healthcare system is fragile and based on limited funds. Availability of poor quality RDT kits could cause havoc. The diagnostic kits analyzed in this study have shown 52% sensitivities for NSP-RDT while 21% for Saliva-RDT, which are not suitable for accurate diagnosis of COVID-19 in Pakistani population. In current scenario continuous monitoring of RDT based COVID-19 kits in Pakistani populations are required to prevent further disease spread.

## Data Availability

The data is available and can be used for the academic or research purposes.

## Abbreviations

SARS-CoV-2: Severe Acute Respiratory Syndrome Coronavirus 2
WHO: World Health Organization
MERS: Middle East Respiratory Syndrome
RDT: Rapid Diagnostic Testing
ACE2: Angiotensin‐Converting Enzyme 2
AT2: Alveolar type 2 progenitor
Ang II: Angiotensin II
Ang 1–7: Angiotensin 1–7
GPCRs: G-Protein-Coupled Receptors
JNK: C-Jun N-terminal Kinase
JAK-STAT: Janus Tyrosine Kinase (JAK)-Signal Transducer and Activator of Transcription
NPS: Nasopharyngeal Swab
RT-PCR: Real-Time Reverse-Transcription Polymerase Chain Reaction
Ct: Cycle Threshold
